# Translation into Portuguese and validation of the Rhinitis Control Assessment Test (RCAT) questionnaire^☆^

**DOI:** 10.1016/j.bjorl.2015.12.011

**Published:** 2016-04-16

**Authors:** Pedro Henrique Fernandes, Fausto Matsumoto, Dirceu Solé, Gustavo Falbo Wandalsen

**Affiliations:** Universidade Federal de São Paulo (UNIFESP), Escola Paulista de Medicina, Departamento de Pediatria, Disciplina de Alergia, São Paulo, SP, Brazil

**Keywords:** Allergic rhinitis, Questionnaires, Control, Validation studies, Rinite alérgica, Questionários, Controle, Estudos de validação

## Abstract

**Introduction:**

The Rhinitis Control Assessment Test (RCAT) is a simple self-administered questionnaire developed to assess control of rhinitis.

**Objectives:**

Translate into Brazilian Portuguese and validate the RCAT.

**Methods:**

The RCAT was translated into Portuguese by two translators and subsequently back-translated into English. It was then applied to 141 adolescents with allergic rhinitis.

**Results:**

The internal consistency of the RCAT was 0.73. The questionnaire scores showed significant correlation with total nasal and extra-nasal symptom scores and nasal peak inspiratory flow (*r*: −0.73, −0.58 and 0.52, respectively; *p* < 0.001) and were significantly different when divided by physician global assessment and total nasal symptom score severity. Cutoff points between 22 and 24 had the higher areas under the ROC curve to identify patients with rhinitis control. Total nasal and extra-nasal symptom scores were significantly different when a cutoff point of 22 was used (median: 4.0 *vs.* 8.0 and 2.0 *vs.* 5.0; *p* < 0.001).

**Conclusions:**

The Brazilian Portuguese version of the RCAT was shown to be a valid and discriminant tool to identify patients with controlled and uncontrolled allergic rhinitis.

## Resumo

**Introdução:**

O *Rhinitis Control Assessment Test* (RCAT) é um questionário simples e autoaplicável desenvolvido para avaliar o controle da rinite.

**Objetivos:**

Traduzir para o Português validar o RCAT.

**Método:**

A tradução do RCAT foi feita por dois tradutores com posterior versão para a Língua Inglesa. O RCAT foi então aplicado de forma transversal a 141 adolescentes com rinite alérgica.

**Resultados:**

A consistência interna do RCAT foi 0,73. As notas totais do questionário se correlacionaram significantemente com os escores de sintomas nasais e extra-nasais e pico de fluxo inspiratório nasal (*r*: -0,73, -0,58 e 0,52, respectivamente; p < 0,001) e foram significantemente diferentes quando separadas pela opinião médica sobre o controle da rinite e pela gravidade dos sintomas nasais. Os pontos de corte entre 22 e 24 foram os com maiores áreas sob a curva ROC para definição do controle da rinite. Os escores de sintomas nasais e extra-nasais foram significantemente diferentes quando os pacientes foram separados pelo ponto de corte de 22 (medianas de 4,0 *vs.* 8,0 e 2,0 *vs.* 5,0; p < 0,001).

**Conclusões:**

A versão em Português do RCAT se mostrou uma ferramenta válida e com bom poder discriminativo para separar pacientes com rinite alérgica controlada e não controlada.

## Introduction

Allergic rhinitis is the most prevalent allergic disease in Brazil. Brazilian data, obtained through the International Study of Asthma and Allergies in Childhood (ISAAC) study showed that the prevalence of rhinitis in children and adolescents ranges from 10% to 47%, depending on the definition used and the age group studied.^1^ Traditionally, allergic rhinitis was considered a disease of minor importance due to its low morbidity and mortality. In the last decade, the importance of allergic rhinitis has been increasingly highlighted, mainly because of its complications, high cost, negative impact on quality of life, and association with other diseases.^2^, ^3^

Currently, the recommendations for the drug management of allergic rhinitis are based on the classification of the disease severity and persistence of symptoms.^2^ These recommendations are easily applicable to patients not under current treatment at the beginning of follow-up, but are less applicable to evaluate changes over time and cannot measure response to treatment. Managing the disease by controlling the magnitude of its symptoms has been demonstrated to be useful and practical in asthma, and is currently the recommended form of management.^4^

A few years ago, a questionnaire was developed to assess the level of rhinitis control called Rhinitis Control Assessment Test (RCAT).^5^, ^6^ This questionnaire, developed in the English language, consists of six questions with five answer levels that comprise a total score. The questions refer to the previous week and address symptoms (nasal congestion, sneezing, and eye tearing), interference with sleep and daily activities, and the individual's personal opinion about symptom control.^5^, ^6^

Evaluation of the original version of RCAT showed that the questionnaire is valid and reliable and can be used for rapid screening of patients with difficult-to-control rhinitis symptoms and as an additional tool in the clinical management of rhinitis.^6^

This study aimed to translate and adapt the RCAT questionnaire into Portuguese language (Brazilian culture), and to validate that translated version.

## Methods

The RCAT questionnaire translation into Portuguese was performed by two different translators with a subsequent back-translation to English and final conciliation of the versions. The resulting version was then applied to ten patients with allergic rhinitis (older than 11 years of age) to evaluate its intellection.

The validation of the translated version of the questionnaire was carried out as an observational, descriptive, analytical, and cross-sectional assessment, applied to patients in a referral allergy outpatient clinic. Adolescents (12–18 years, both genders), with documented diagnosis of allergic rhinitis for at least six months were invited to participate in the questionnaire validation. Only patients with proven allergic sensitization to at least one inhaled allergen, demonstrated by skin prick test or specific serum IgE tests performed in the last two years participated in the study.

Those with a history of symptoms consistent with infectious diseases of the upper airways in the last 15 days, as well as those with neuropathy, cognitive deficits, and structural changes of the upper airways (clinical assessment) were not invited to participate in the study.

The patients answered the translated version of RCAT, which consisted of six questions related to symptoms experienced in the previous week. Each question received scores ranging from 1 to 5 points according to the frequency of reporting, with score 5 for “never,” 4 for “rarely,” 3 for “sometimes,” 2 for “often,” and 1 for “very often”. The final score (RCATT), given by the sum of all questions, could range from six to 30 points.^6^ According to the total score obtained, patients were divided into two groups according to the original questionnaire validation: controlled (≥22 points) and uncontrolled (<22 points).^6^

Additionally, patients were evaluated through the Nasal Symptom Score (NSS) and the Extra-Nasal Symptom Score (ENSS). The NSS was related to the week prior to the assessment, assigned by the patient (ranging from 0 to 3) for rhinorrhea, nasal obstruction, nasal itching, sneezing, and postnasal drip, and calculated by adding the scores for the five symptoms. The severity of the clinical condition was classified according to the NSS, being considered mild (NSS 0–5), moderate (NSS 6–10), or severe (NSS 11–15).^6^ The ENSS, also related to the week prior to the assessment, was measured by addressing the following symptoms: eye tearing, eye itching, pharyngeal pruritus, and ocular hyperemia, which were scored according to the NSS (maximum 12).

An objective assessment of nasal function was performed by measuring the Peak Nasal Inspiratory Flow (PNIF) using specific equipment (In-Check^®^; Clement Clarke, England). During the procedure, patients were instructed to perform deep inspirations up to total lung capacity, keeping the lips tightly closed. The maximum flow rate was read by the cursor in liters per minute. At least three measurements were performed and the best measurement among the three was recorded, with a variation of less than 10%.^7^

All patients underwent medical assessment before the questionnaires were applied, in which any medications being used and associated comorbidities were recorded. Medical opinion was requested about the patients’ nasal symptom control, and the physician's subjective classification noted as controlled, partially controlled, or uncontrolled. Similarly, the physician reported his/her opinion regarding adherence or not to the proposed treatment in previous consultations, according to data obtained at the interview.

The study was approved by the Research Ethics Committee of the institution (protocol n. 282867) and an informed consent form was obtained from all participants and their parents/guardians.

Sample size calculation was performed based on the correlation coefficients found between the scores of the original questionnaire and the clinical scores ranging from 0.3 to 0.6.^6^ Thus, estimating a minimum *r* of 0.3, at least 116 patients would be necessary to ensure significant correlation with a power of 95% and *p* = 0.05.

The constructive validation of the translated version of RCAT was performed by comparing the scores obtained with the NSS, ENSS, and PNIF, using Spearman's correlation test. The discriminatory capacity was assessed by comparing the RCAT scores according to the medical classification of rhinitis control and severity of nasal symptoms using non-parametric tests (Mann–Whitney and Kruskal–Wallis). The internal consistency of RCAT was assessed by Cronbach's alpha. Receiver operating characteristic curves were constructed to establish cutoff scores, according to the medical opinion about disease control (controlled rhinitis *vs.* partially controlled and uncontrolled rhinitis).

## Results

**Figure 1:**
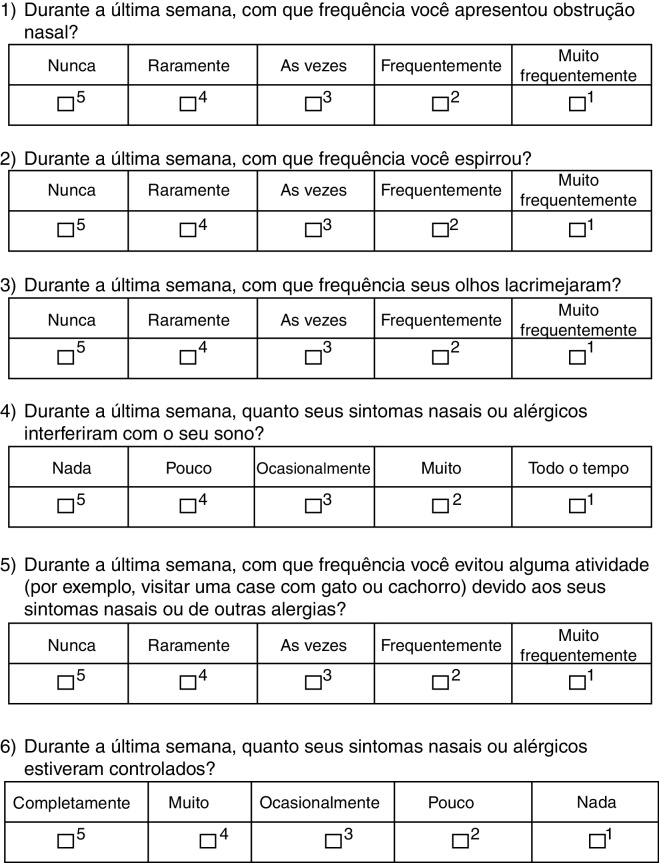
Translated version of the Rhinitis Control Assessment Test (RCAT) into the Portuguese language (Brazilian culture).

The RCAT version that was translated into Portuguese (Brazilian culture) is shown in Fig. 1. There were no significant differences between the two translations into Portuguese, and therefore the final consolidation of the translated RCAT and the English version did not result in any changes to the original tool. At the initial assessment, the questionnaire was easily understood by the adolescents and it was quickly completed.

A total of 141 adolescents aged between 12 and 18 years (median 13 years), participated in the validation phase, of whom the majority was male (74%) and white (47%). The median of years with nasal symptoms was 8 years (interquartile range [IQR]: 5–11 years). All patients attended school and were literate. The internal consistency of the questionnaire measured by Cronbach's *α* was 0.73.

Regarding the medical opinion about the control of patients’ nasal symptoms, 53 (38%) were classified as controlled, 51 (36%) as partially controlled, and 37 (26%) as uncontrolled. According to the medical opinion, 106 (75%) patients were considered adherent and 35 (25%) were non-adherent to treatment.

The NSS median was 5 (IQR: 3–8), the ENSS median was 3 (IQR: 1–6), and PNIF median was 130 L/min (IQR: 100–150 L/min). According to the NSS score, mild symptoms were observed in 78 patients (55%), moderate in 50 (36%), and severe in 13 (9%). The RCAT scores ranged between 10 and 30, with a median of 22 (IQR: 19–26).

**Figure 2:**
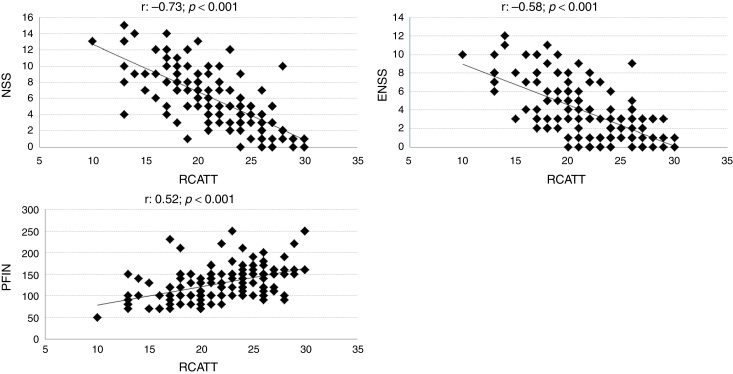
Correlation between total Rhinitis Control Assessment Test (RCAT) scores (RCATT) and Nasal Symptom Score (NSS), Extra-Nasal Symptom Score (ENSS), and Peak Nasal Inspiratory Flow (PNIF) values.

The correlation coefficients of RCAT with the NSS, ENSS and PNIF were respectively −0.73; −0.58; and 0.52 (*p* < 0.001). Individual values are shown in Fig. 2.

**Figure 3:**
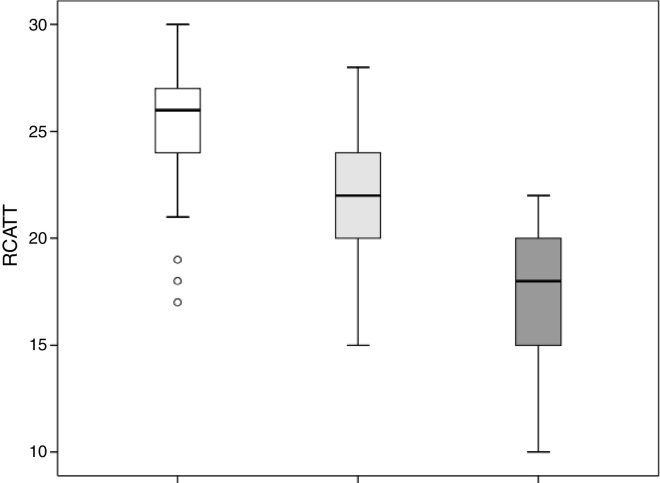
Total score values of Rhinitis Control Assessment Test (RCAT) discriminated by the medical opinion about the control of nasal symptoms as controlled (white), partially controlled (light gray), and uncontrolled (dark gray).

**Figure 4:**
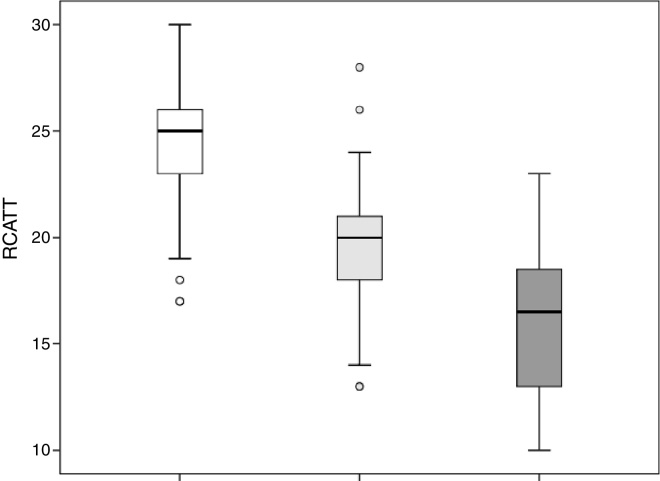
Total score values of Rhinitis Control Assessment Test (RCAT) discriminated by the severity of nasal symptoms as mild (white), moderate (light gray), and severe (dark gray).

The RCAT scores were significantly different (*p* < 0.001) when separated by the medical opinion about nasal symptom control (Fig. 3) and nasal symptom severity (Fig. 4).

Sensitivity, specificity, and area under the ROC curve of RCAT for different cutoff points to define rhinitis control are shown in Table 1. The cutoff point of 22, defined in the original questionnaire validation, showed a sensitivity of 89% and specificity of 66% in the definition of allergic rhinitis symptom control. The cutoff scores with larger areas under the ROC curve were 23 and 24 points.

**Table 1:** Sensitivity, specificity, and area under the receiver operating characteristic (ROC) curve of different Rhinitis Control Assessment Test (RCAT) cutoffs to define rhinitis control.

Cutoff point	Sensitivity (%)	Specificity (%)	Area under the ROC curve
≥25	60.4	87.5	0.739
≥24	79.2	79.5	0.794
≥23	84.9	73.9	0.794
≥22	88.7	65.9	0.773
≥21	94.3	56.8	0.756
≥20	94.3	42.0	0.682
≥19	96.2	35.2	0.657

**Figure 5:**
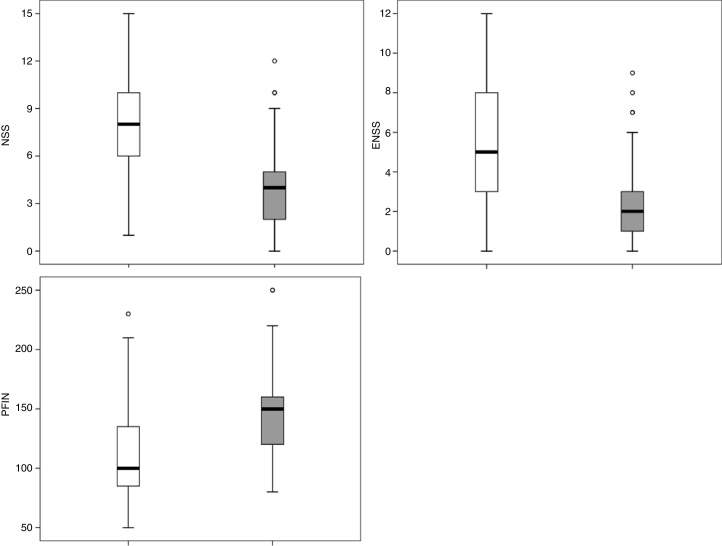
Score values of Nasal Symptom Score (NSS), Extra-Nasal Symptom Score (ENSS) and Peak Nasal Inspiratory Flow (PNIF) values of patients classified as controlled (gray; total score values of Rhinitis Control Assessment Test (RCAT) ≥22), or uncontrolled (white; RCATT <22).

Applying the RCAT cutoff score of 22 points, 77 patients were defined as controlled and 64 as uncontrolled. The NSS, ENSS, and PNIF were significantly different between these two groups (*p* < 0.001) with medians of, respectively: 4.0 *vs*. 8.0; 2.0 *vs.* 5.0; and 150 L/min *vs*. 100 L/min (Fig. 5).

## Discussion

Tools or scores have frequently been used in the management of several chronic diseases, such as asthma and chronic hives.^8^, ^9^ These tools can have many applications and are used both to screen patients in primary care settings and to assist in medical management by specialists.

Several questionnaires are available for the assessment of patients with nasal diseases. Among these, those developed to assess the quality of life in patients with specific diseases, such as allergic rhinoconjunctivitis^10^ and chronic rhinosinusitis/nasal polyposis (SNOT-22),^11^ translated and validated into the Portuguese language (Brazilian culture), are noteworthy.^12^, ^13^ Other questionnaires assess certain symptoms, such as nasal obstruction (NOSE),^14^ or a combination of diseases, such as rhinitis and asthma.^15^, ^16^ To the best of the present authors’ knowledge and that of other authors,^5^ the RCAT was the first tool of this type to be developed for the assessment of allergic rhinitis control.

The RCAT was developed as a simple, concise, and self-administered tool to assess rhinitis control.^5^ The items in the questionnaire were selected with the help of groups of patients and physicians. Initially, 26 questions were identified, separated into five areas: symptoms, interference in activities, limitations, rhinitis control, and medication use.^5^ After application to a large group of patients, this initial version of the questionnaire went through an assessment process in which the most relevant questions were identified through logistic regression analyses, and the final version comprised six questions.^5^

In this study, some important properties of the translated version of RCAT were assessed. The reliability was evaluated by its internal consistency, with an acceptable value (>0.7) as demonstrated by Cronbach's *α* coefficient.

In the constructive validation, the overall questionnaire scores (RCAT) showed a strong correlation with the nasal symptom score (NSS). The correlation coefficient found (−0.73) was higher than that observed when the original version of RCAT was validated (−0.57).^6^ Unlike the NSS, the RCAT addresses additional aspects besides rhinitis symptoms, such as the interference of rhinitis with sleep and daily activities and, thus, some degree of disagreement between these tools was expected. Differences in the tools are more marked in relation to the ENSS and the objective measurement of nasal function (PNIF). Nevertheless, moderate correlations were observed between them (Fig. 2).

The capacity of the translated version of RCAT to discriminate patients according to the degree of rhinitis control is easily observed in Figure 3, Figure 4. These figures show that the final scores of the RCAT are clearly different when patients are separated by the medical opinion about disease control or the severity of the NSS.

The identification of the best RCAT cutoff to discriminate controlled patients from those with problems in allergic rhinitis control may vary according to the purposes and aims of its application, and it is possible to choose cutoff points with greater sensitivity or greater specificity. In the original validation of RCAT, the cutoff point with the largest area under the ROC curve was 22 points (AUC = 0.689). This cutoff point also showed a large area under the ROC curve in the validation of the translated version (AUC = 0.773; Table 1). When this cutoff point was applied to the group of studied patients, significant differences were observed for the NSS, ENSS, and PNIF values (Fig. 5). Among the present patients, however, the cutoff points of 23 and 24 showed larger areas under the ROC curve, particularly due to the biggest gain in specificity without significant losses in sensitivity (Table 1).

The group of patients evaluated in our study differed in some aspects from the group evaluated in the original validation of RCAT; the most relevant aspects were the age group and the diagnosis. In the original validation of RCAT, only patients aged 18 years or older could participate, whereas the present study included only adolescents aged 12–18 years. The present patients had persistent allergic rhinitis, exclusively, unlike the original validation, comprising patients with perennial allergic, seasonal, and non-allergic rhinitis.^6^

Further studies are necessary to evaluate other properties of the translated version of RCAT, already studied in the original version of the questionnaire, such as its reproducibility and the clinically relevant minimum difference.

## Conclusions

In conclusion, this study presented a version of the RCAT rhinitis control questionnaire translated into the Portuguese language (Brazilian culture). It was observed that this version of the questionnaire is easily understood by adolescents with allergic rhinitis, and it is a valid tool with good discriminatory power to differentiate controlled patients from those non-controlled.

## Conflicts of interest

The authors declare no conflicts of interest.
